# The effect of malaria and anti-malarial drugs on skeletal and cardiac muscles

**DOI:** 10.1186/s12936-016-1577-y

**Published:** 2016-11-02

**Authors:** Mauro Toledo Marrelli, Marco Brotto

**Affiliations:** 1 Department of Epidemiology, School of Public Health, University of São Paulo, Avenida Dr. Arnaldo 715, São Paulo, SP 01246-904 Brazil; 2 Bone-Muscle Collaborative Science, College of Nursing & Health Innovation, University of Texas-Arlington, 501 S. Nedderman Drive, Life Science Building, Suite 436/437, Arlington, TX 76019-0498 USA

**Keywords:** Malaria, Skeletal muscles, Cardiac muscle, Fatigue, Anti-malaria drugs

## Abstract

Malaria remains one of the most important infectious diseases in the world, being a significant public health problem associated with poverty and it is one of the main obstacles to the economy of an endemic country. Among the several complications, the effects of malaria seem to target the skeletal muscle system, leading to symptoms, such as muscle aches, muscle contractures, muscle fatigue, muscle pain, and muscle weakness. Malaria cause also parasitic coronary artery occlusion. This article reviews the current knowledge regarding the effect of malaria disease and the anti-malarial drugs on skeletal and cardiac muscles. Research articles and case report publications that addressed aspects that are important for understanding the involvement of malaria parasites and anti-malarial therapies affecting skeletal and cardiac muscles were analysed and their findings summarized. Sequestration of red blood cells, increased levels of serum creatine kinase and reduced muscle content of essential contractile proteins are some of the potential biomarkers of the damage levels of skeletal and cardiac muscles. These biomarkers might be useful for prevention of complications and determining the effectiveness of interventions designed to protect cardiac and skeletal muscles from malaria-induced damage.

## Background

Malaria remains as the most important human infectious diseases in the World, with around 214 million cases a year, and an astounding 438,000 deaths resulting from this disease alone [1]. About 3.2 billion people live in areas at risk of malaria. Populations of the poorest countries are the most vulnerable. More than 800 children die of malaria each day in Africa, according to a report of the World Health Organization [1] (one child every 2 min). In addition, non-endemic countries have reported hundreds of imported malaria cases [2]. In 2013, 1727 cases of malaria were reported in the USA, and the US Armed Forces Command has named malaria as the number 1 enemy, because of the high exposure levels of soldiers in the highly endemic areas where they are frequently deployed on a range of missions [3].

The human malaria is caused by five different species and *Plasmodium falciparum* can result in severe malaria and death if adequate treatment is not provided quickly. The pathogenesis mechanisms of several diseases caused by protozoan and nematode parasites have shown to cause detrimental effect on cardiac and skeletal muscles (i.e., Chagas disease, toxoplasmosis, trichinosis, leishmaniosis, and malaria) [4–7]. Parasitic infestations by *Trypanossoma cruzi*, *Toxoplasma gondii* and *Trichinella spiralis* cause cardiomyopathy in the immunocompetent and immunocompromised patients. Besides those parasites, *Plasmodium falciparum* infection can also cause parasitic coronary artery occlusion [8].

Malaria pathogenesis is a process by which malaria parasites cause illness, abnormal function, or damage in their animal or human hosts. “Uncomplicated” malaria entails a series of recurring episodes of chills, intense fever, and sweating and often includes other symptoms such as headache, malaise, fatigue, body aches, nausea, and vomiting. In some cases, and especially in groups, such as children and pregnant women, the disease can progress to “severe malaria,” including complications, such as cerebral malaria/coma, seizures, severe anaemia, respiratory distress, kidney and liver failure, cardiovascular collapse, and shock [9–16]. Skeletal muscle is the largest organ-system of the human body and, as expected, malaria significantly affects skeletal muscle function and metabolism. In fact, among the above-mentioned malaria symptoms, many of them can be attributed to dysfunction of the skeletal system. This article reviews the current knowledge about the involvement of malaria disease and the anti-malarial drugs used in its treatment effecting skeletal and cardiac muscles.

## Malaria affecting skeletal muscles

The detrimental effects of the causing malaria agents on skeletal muscles in animals and humans are well known [11, 16–20]. The main pathogenic mechanism in severe malaria is microvascular sequestration of parasitized red blood cells, decreasing oxygen delivery, leading to obstructed blood flow and tissue hypoxia [20]. The skeletal muscle microvascular function and its oxygen consumption is significantly impaired in malaria in the proportion of the disease severity and oxygen consumption in severe malaria reduces similarly as in sepsis patients [20].

Several case reports have been published regarding malaria effects on skeletal muscles [11, 17]. Skeletal muscle necrosis was reported in a patient with severe falciparum malaria, probably due to sequester of infected erythrocytes, causing microcirculatory obstruction [10]. Rhabdomyolysis, a serious syndrome directly or indirectly caused by muscle injury or death, can lead to complications, such as kidney failure due to intense myoglobinuria, have been commonly reported in malaria patients [18].

The injured skeletal muscles has biomarkers relating with severity of falciparum malaria infection [16, 21, 22], and the sequestration of infected red blood cells has been pointed out as the cause for these processes. Pronounced deviation in normal serum levels of creatine kinase (CK) have been also reported in malaria patients, affecting skeletal muscles [16, 21]. CK is an enzyme involved in the synthesis and use of energy-providing molecules, and it is predominantly found in cells of cardiac and skeletal muscles. A longitudinal study suggested that falciparum malaria is associated with skeletal muscle damage that increases during the course of the disease and directly associates with abnormalities in CK levels [21]. In addition, the inflammatory characteristic of *Plasmodium* parasites increases cytokines levels (such as tumour necrosis factor, TNF) in combination with the formation of highly damaging free radicals [16], which could be considered as a potential important mechanism of damage and muscle weakness.

**Fig. 1:**
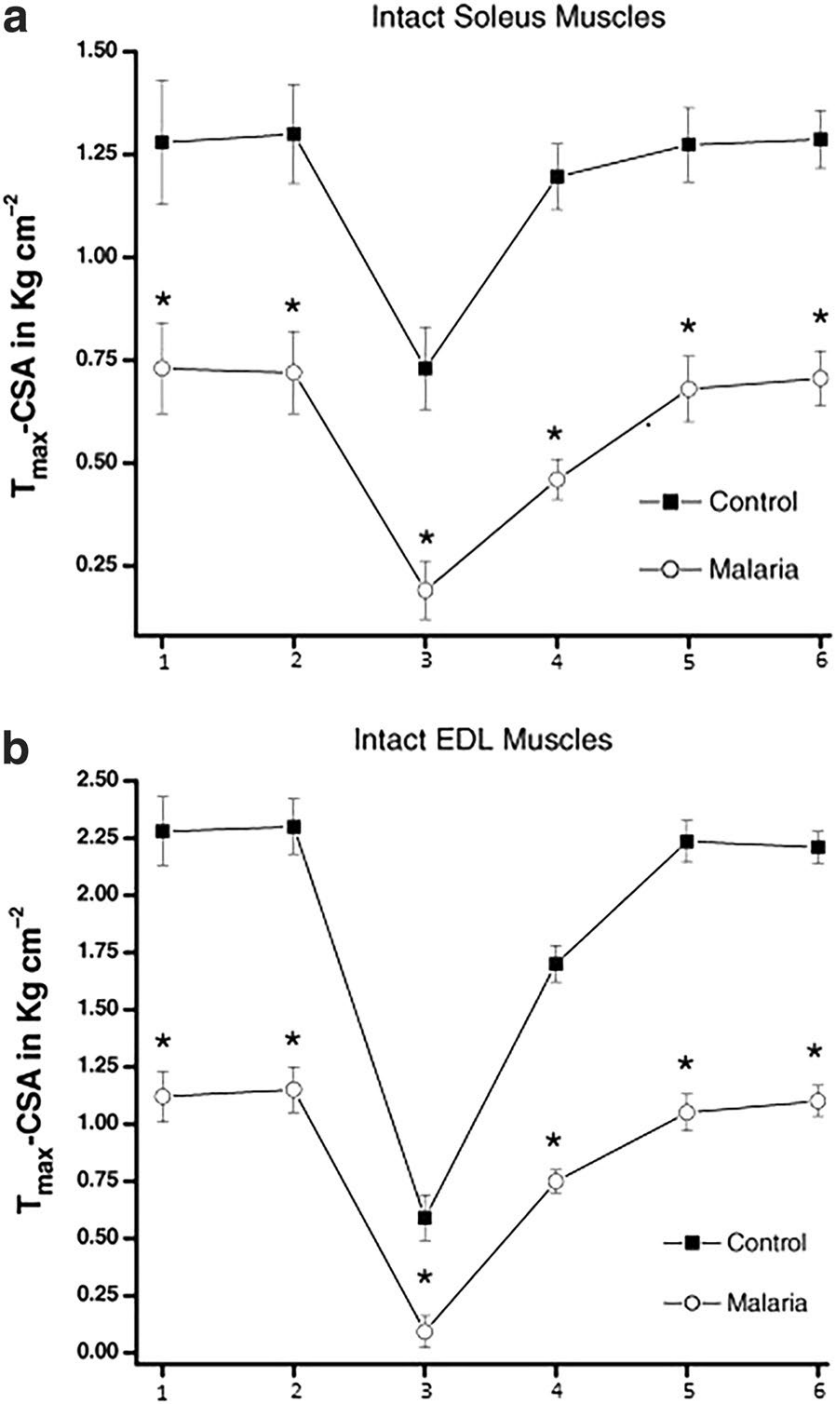
Summarized maximum tetanic force (*Tmax*) data at specific protocol time-points, for intact (**a**) Soleus and (**b**) EDL muscles. Data were normalized to the cross-sectional area produced by intact muscles from control and malarial mice (n = 10). Points: (*1*) start 20 min equilibration, (*2*) end 20 min equilibration, (*3*) end of fatigue, (*4*) 30 min recovery, (*5*) 45 min recovery +20 mM caffeine, (*6*) 60 min recovery +20 mM caffeine This figure is modified from Brotto et al. [19]

**Fig. 2:**
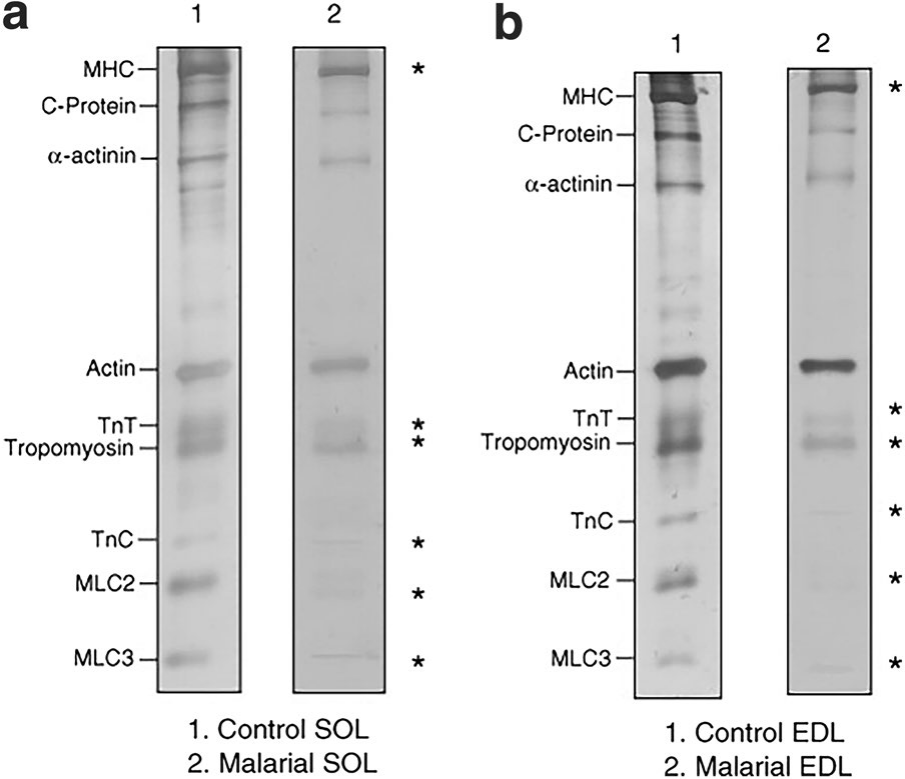
Representative silver-enhanced SDS-PAGE of 20 Triton-skinned fibers from control (*lane 1*) and 18 malarial fibers (*lane 2*) from SOL (**a**) and EDL (**b**) muscles: myosin heavy chain (MHC), troponin T (TnT), tropomyosin, troponin C (TnC), myosin light chain type II (MLC2) and myosin light chain type III (MLC3). This figure is from Brotto et al. [19]. Density levels for control bands were normalized to 100% and compared to the density of malarial fibers. *Asterisks* show significant difference found between fibers densities tested with Kruskal–Wallis one-way analysis of variance

Much lower levels of RNA and protein contents were found in skeletal muscles (such as soleus muscles) than in non-muscle tissues of malaria infected rats and, when compared with non-infected rat controls [23], suggesting overall increase in protein degradation or enhanced catabolism. Corroborating with these findings, Brotto et al. [19] demonstrated that in mice infected with *Plasmodium berghei*, both the extensor digitorum longus (EDL) and soleus (SOL) muscles produced approximately half of the normal contractile force, fatigued significantly more, and recovered significantly less from fatigue (Fig. 1). This study helped to begin the understanding of these detrimental effects of malaria on skeletal muscles through the utilization of skinned muscle fibers that revealed direct effects on the contractile machinery itself. These effects were associated with significant decrease in content of key contractile proteins (reduced from 15 to 45%) in the skinned fibers from malaria mice (Fig. 2) [19], in agreement with findings from other studies of the presence of muscle specific proteins in the circulation [16, 21–24]. It is important to note that malaria may also affect the activity of infected vertebrates, which may contribute to some of the loss of muscle protein in less active rats.

## Effects of malaria on cardiac muscle

Few studies have been focused on cardiac effect in severe malaria [25–27] despite serious symptoms of coronary complications have been observed in severe malaria patients. Although, few electrocardiographic reports of malaria patients have been published [28–33], their results have shown even some cases of deaths related to cardiac arrhythmias in severe malaria [32].

Circulation levels of cardiac proteins, such as troponin T (TnT), myoglobin and creatine kinase (CK), which are biomarkers of myocardial injury, increases with the severity of malaria, indicating myocardial impairment in complicated falciparum malaria [24, 25]. In a case–control study, myoglobin, CK, plasma levels of N-terminal pro-brain natriuretic peptide (NT-proBNP) and heart-type fatty acid-binding protein (H-FABP) were compared in 400 African children with severe and mild falciparum malaria, showing that children suffering from severe malaria and children who died due to malarial infection, exhibited high to very high levels of cardiac biomarkers, respectively [24]. In another case–control study with 63 malaria patients showed that elevated levels of NT-proBNP and H-FABP indicated myocardial impairment in complicated but not in uncomplicated falciparum malaria [25].

Cardiac troponin T is the most sensitive biomarker for detection of minimal myocardial damage [34]. Release of these troponins can occur when myocytes are damaged by a variety of conditions such as inflammation, trauma, exposure to toxins and necrosis because of occlusion of a coronary vessel [35, 36]. Although severe, myocardial cell damages detected by high troponin T levels are rare events, as shown by a retrospective case study of 161 stored sera of malaria falciparum patients [34].

By analyzing gene expression of cardiomyocytes treated with purified *P. falciparum* glycosyl phosphatidylinositol (GPI), which act as a toxin in the malaria pathogenesis, up-regulated genes related to apoptosis and myocardial damages were found, indicating that *P. falciparum* GPI can also induce cardiomyocyte apoptosis [37]. In the same study, a falciparum infected patient presented with heart failure with typical signs of cardiac myocyte apoptosis, suggesting that his complications could have been caused by the presence of this toxin.

Some case reports have also suggested an association of *Plasmodium vivax* infection and the appearance of cardiac complications, with diagnosis of acute coronary syndrome, tachycardia, arrhythmia and myocardial failure. However, there is the possibility of anti-malarial therapy being the cause of these cardiac complications [38–40].

In a study using rodent malaria models, irreversible lesions were observed in several organs, including heart, of mice infected with *Plasmodium chabaudi*, *Plasmodium vinckei petteri* and *Plasmodium yoelii nigeriensis*, following acute and chronic malaria [41].

## Anti-malarial drugs and their effects on cardiac and skeletal muscles

Treatment for malaria disease can only be initiated after making the correct diagnosis. Once made, appropriate anti-malarial treatment must be initiated immediately. Treatments are guided by three main factors: (i) the infecting species of *Plasmodium*, (ii) the clinical condition of the patient, and (iii) the susceptibility of the parasites to the anti-malarial drugs determined by the geographic area where the infection was acquired and the previous use of the medicines.

Most anti-malarial drugs used are active against the blood parasite stages (the forms that causes the symptoms) and include mainly: chloroquine, mefloquine, quinine, quinidine, doxycycline, clindamycin and artemisinins. Widely-used anti-malarial drugs have a limited clinical lifespan due to increasing parasite resistance development. For instance, chloroquine, the drug of choice most used in Africa to combat malaria is no longer effective against the disease in several of the malaria endemic areas. Artemisinin-based combination therapy is currently the recommended treatment against falciparum malaria, in many parts of the world. However, there is serious concern that malaria parasites are developing resistance to this treatment. With parasite resistance continuously rising, anti-malarial drug discovery requires strategies to decrease the time of delivering a new anti-malarial drug while simultaneously increasing the efficacy of existing treatments, developing alternative treatments [42], as well putting in place preventative measures, such as bed nets. In addition to causing serious whole body side effects, these drugs also have the ability to directly affect skeletal and cardiac muscles [43, 44].

Chloroquine is used in treatment and prophylaxis of malaria in areas where malaria is known to be sensitive to its effects. Prolonged administration of it can cause heart block and progressive myopathy [42]. It has also been reported as cause of toxicity, mostly in the retina [45, 46]. Chloroquine is commonly used in the treatment of autoimmune disorders such as rheumatoid arthritis and systemic lupus erythematosus. Chloroquine increases the pH in lysosomal lumens, causing the disruption of lysosomal degradation of proteins [47]. Ikezoe et al. [44], demonstrated that chloroquine triggers the disruption of lysosomal enzymes, inducing amyloid-β accumulation and endoplasmic reticulum (ER) stress. Amyloid-β causes skeletal muscle fiber degeneration, activating autophagy in skeletal muscles of rats.

Cases of neuromyopathies and cardiomyopathies due to chronic chloroquine intoxication have been reported [48], in long-term therapies of autoimmune disorders (mainly in systemic lupus erythematosus) and during the use of chloroquine in malaria prophylaxis. Due to its lower toxicity comparing with chloroquine, hydroxychloroquine is preferentially used today.

Quinine, a drug of choice in areas of chloroquine resistance, can cause serious toxic effects, such as arrhythmia, angina and hypotension, circulatory defect and shock [15, 49]. Case reports of severe malaria patients presenting arrhythmia during a treatment with quinine have been published [50]. A fatal ventricular fibrillation was reported in a malaria patient, with no heart disease history, probably due to the toxicity of the administrated quinine during the treatment [51]. Although rare, the reports show that more attention has to be given during malaria treatment due to toxicity of quinine.

Another malaria drug, artesunate, has been used as alternative to treat malaria with minimal toxicity. However, it has been shown to affect airway smooth muscles, inhibiting the proliferation of human cultured airway smooth muscle cells, causing hyperplasia and hypertrophy [52], which leads to obstruction of the airways [53].

## Conclusion

Long-term impacts of malaria include death, disability, and significant socioeconomic burden on societies where the disease is prevalent. A better understanding of the biological processes underlying the progression of infection to disease is urgently needed to reduce the morbidity and mortality of malaria.

As many diseases caused by protozoan parasites, malaria has shown to cause detrimental effect on cardiac and skeletal muscles [5–7, 11]. Malaria in humans leads to muscle weakness, muscle fatigue, respiratory distress, kidney and liver failure, and can lead to cardiac myopathies. These severe complications can also be linked to skeletal muscle damage, besides the more readily recognized effects on erythrocytes.

**Fig. 3:**
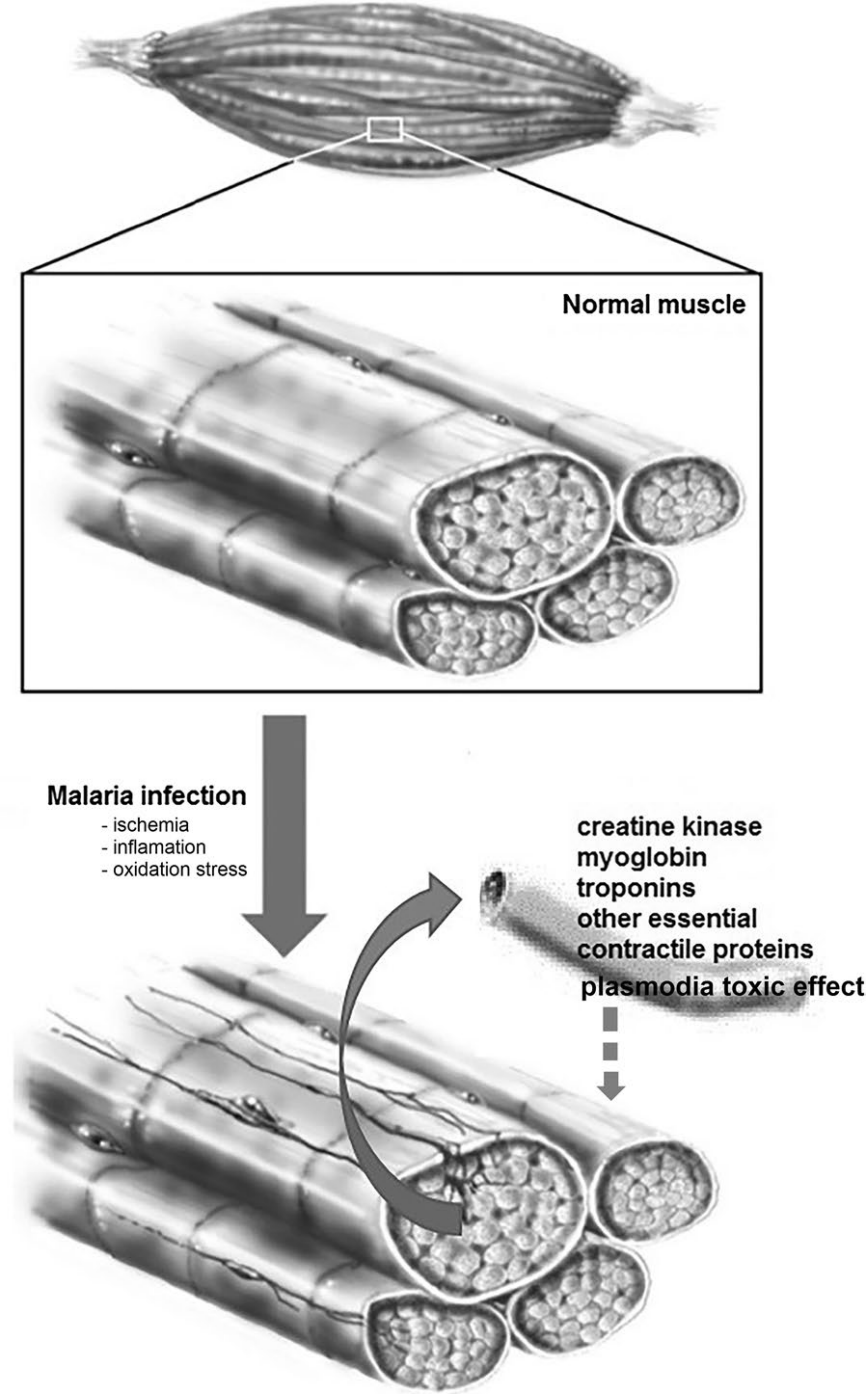
Hypothetical model of malaria infection. Malarial infection leads to ischemia due to sequestration of red blood cells, inflammation and oxidative stress, which in turn damages skeletal and cardiac muscles. Creatine kinase, myoglobin, troponins and other essential contractile proteins released into the blood stream. While these factors might be used as biomarkers for muscle damage caused by malaria parasites or for the progression or the severity of the disease, they might as indicated by the dotted line create a feedback loop whereas they induce additional damage. Furthermore the toxic effect of *Plasmodium* is also released in the blood and can re-circulate to induce further damage This figure was modified from Abreu et al. [54]

A hypothetical model is shown in Fig. 3, which predicts that malaria infection causes muscle damage due to the combination of ischemia, inflammation, and oxidative stress. Injured muscle fibers release significantly larger amounts of creatine kinase, myoglobin and other essential contractile proteins into the blood, such as troponins, which helps to explain how these proteins might leak into the bloodstream, and the associated muscle weakness and muscle fatigue in malaria patients. These released proteins/factors might create a feedback loop whereas they might lead to additional membrane and cellular damage. An intriguing possibility is that the early detection of these proteins in the blood of malaria patients could make them useful as biomarkers for muscle damage caused by malaria parasites. Increased serum levels of proteins resulting from muscle damage may also help explain some of the damage to other organs such as the liver and kidney in these patients. In addition, the toxic effect of *Plasmodium* species, increasing cytokines levels and free radicals could also be considered as potential mechanism of damage and muscle weakness. Together, this information could be used for improved monitoring of disease progression and development of specific interventions for the protection of cardiac and skeletal muscles against the damaging effects of both the infection and the treatment.

Some of the findings reviewed in this article can help explain many of the symptoms in humans infected with malaria. Malaria infection induces a combination of inflammation and oxidative damage in skeletal and cardiac muscles leading to the enhanced degradation of key contractile proteins, which in turn is responsible for the compromised muscle function. Therefore, physiological key questions need to be answered: (i) What is the mechanism of direct damage to the contractile machinery? (ii) What are the genetic signaling pathways/networks regulating these modifications? (iii) Can these detrimental effects be counteracted with specific exercise training modalities, antioxidants therapy, dietary interventions or new drugs rationally designed to prevent the muscle damage?

These questions and many more and the large toll exerted by malaria clearly shows the need for investigation to determine the cellular and molecular mechanisms of malaria induced muscle damage.
